# Retracted: Protective Effects of Soy Oligopeptides in Ultraviolet B-Induced Acute Photodamage of Human Skin

**DOI:** 10.1155/2020/8265810

**Published:** 2020-08-28

**Authors:** Oxidative Medicine and Cellular Longevity

*Oxidative Medicine and Cellular Longevity* has retracted the article titled “Protective Effects of Soy Oligopeptides in Ultraviolet B-Induced Acute Photodamage of Human Skin” [1]. The article was found to contain duplicated images as follows:

- The fourth image in Figure 6A is the same as the third image in Figure 7B
- The third image in Figure 6C is the same as the first image in Figure 6D, if slightly rotated anticlockwise

The authors apologized for this mistake and stated that it was out of carelessness and they asked to correct the duplicated images. However, the Editorial Board recommended retraction. The authors agree to retraction.
